# Supplementary material for: Stress in the city: meta-analysis indicates no overall evidence for stress in urban vertebrates
Source: Proc Biol Sci. 2020 Oct 7;287(1936):20201754. doi: 10.1098/rspb.2020.1754 (PMC7657868; doi:10.1098/rspb.2020.1754)
Supplement: Supplementary results [file rspb20201754supp1.docx]

**Stress in the city: meta-analysis indicates no overall evidence for stress in urban vertebrates**

**Supplementary information:**

**Figure S1**. PRISMA Flow diagram reporting the number of studies identified and excluded during the literature screening process.


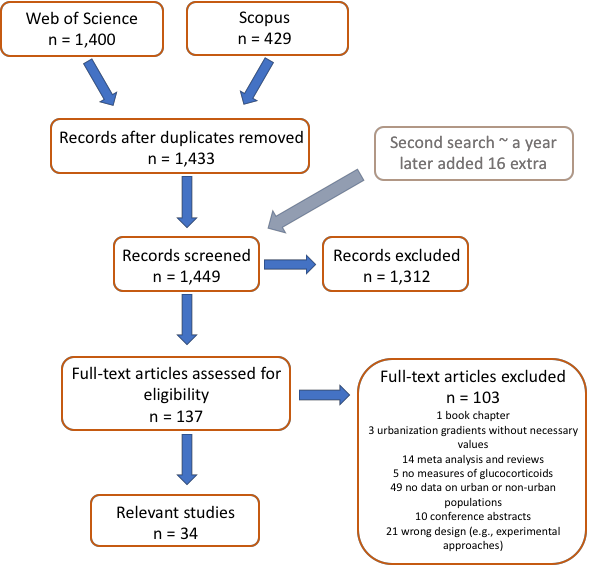


**Figure S2**. Funnel plots of the meta-analytic residuals and the precision (1/SE) for the baseline and stress-induced glucocorticoids.

**Figure S3**. Correlation plot between each effect size and the human population size of the corresponding city (log-transformed) for baseline and stress-induced glucocorticoid levels in all taxa.

**Table S1.** Summary of the number of species, studies and effect sizes for each level within each moderator for: a) baseline glucocorticoids; and b) stress-induced glucocorticoids that included all taxa. The levels excluded from our meta-regressions and the reasons for this are presented in grey. In general, we excluded categories that had < 2 studies or < 2 species, and < 6 effect sizes.

| a) Baseline glucocorticoids: All taxa | | |  |  |
| --- | --- | --- | --- | --- |
|  | **Species** | **Studies** | **Effect size** | **Reason for exclusion** |
| ***Sex*** |  |  |  |  |
| Female | 10 | 10 | 24 |  |
| Male | 16 | 19 | 59 |  |
| ***Life stage*** |  |  |  |  |
| Juveniles | 3 | 4 | 8 |  |
| Adults | 25 | 31 | 100 |  |
| ***Season*** |  |  |  |  |
| Breeding | 12 | 18 | 47 |  |
| Moulting | 4 | 1 | 8 | Single study, no variation |
| Non-breeding | 14 | 10 | 30 |  |
| ***Source of hormone*** |  |  |  |  |
| Total Blood | 13 | 19 | 46 |  |
| Free Blood | 5 | 2 | 14 |  |
| Feather | 3 | 3 | 7 |  |
| Fecal | 11 | 10 | 28 |  |
| Hair | 2 | 2 | 10 |  |
| Water | 1 | 1 | 3 | Single study and species, no variation |
|  |  |  |  |  |
| b) Stress-induced glucocorticoids: All taxa | | | |  |
|  | **Species** | **Studies** | **Effect size** | **Reason for exclusion** |
| ***Sex*** |  |  |  |  |
| Female | 4 | 4 | 5 | Number of effect sizes below our threshold |
| Male | 9 | 11 | 40 |  |
| ***Life stage*** |  |  |  |  |
| Juveniles | 2 | 2 | 6 | Insufficient variation within juveniles for other moderators, |
|  |  |  |  | so we performed our meta-regressions based on adults only |
| Adults | 14 | 18 | 48 |  |
| ***Season*** |  |  |  |  |
| Breeding | 7 | 11 | 24 |  |
| Moulting | 4 | 1 | 8 | Single study, no variation |
| Non-breeding | 7 | 4 | 13 |  |
| ***Source of hormone*** |  |  |  |  |
| Total Blood | 11 | 15 | 36 |  |
| Free Blood | 5 | 2 | 14 |  |
| Feather | 0 | 0 | 0 |  |
| Fecal | 2 | 2 | 2 | Number of effect sizes below our threshold |
| Hair | 0 | 0 | 0 |  |
| Water | 1 | 1 | 2 | Single study and species, no variation |

**Table S2**. Model coefficients and lower (L.) and upper (U.) 95% credible intervals (CI) for the effect (*g*) of habitat (urban), and for each of the moderators, on baseline glucocorticoid levels of birds. The table on the right shows the heterogeneity explained by each of the random effects for the full intercept-only model.

| **Baseline glucocorticoid levels** | | |  |  |  |  |  |  |
| --- | --- | --- | --- | --- | --- | --- | --- | --- |
| **Birds only** |  |  |  |  |  |  |  |  |
|  | **Est** | **L. CI** | **U. CI** |  |  | ***I^2^* Est.** | **2.5 % CI** | **97.5 % CI** |
| *Intercept-only model* | |  |  |  | Species ID | 0.395 | 0.222 | 0.562 |
| *All data, N = 68* |  |  |  |  | Study ID | 0.000 | 0.000 | 0.000 |
| Intercept | 0.105 | -0.224 | 0.435 |  | Study/Group | 0.000 | 0.000 | 0.000 |
| *Excluding outlier, N = 67* | |  |  |  | Total | 0.754 | 0.673 | 0.822 |
| Intercept | 0.088 | -0.235 | 0.411 |  |  |  |  |  |
| *One effect size per group, N = 53* | | |  |  |  |  |  |  |
| Intercept | 0.067 | -0.276 | 0.409 |  |  |  |  |  |
| *Moderator: hormone source* | | |  |  |  |  |  |  |
| *All data, N = 60* |  |  |  |  |  |  |  |  |
| Intercept | -0.314 | -1.245 | 0.617 |  |  |  |  |  |
| Faeces | 0.385 | -1.076 | 1.847 |  |  |  |  |  |
| Free Blood | 0.839 | -0.254 | 1.933 |  |  |  |  |  |
| Total Blood | 0.458 | -0.561 | 1.477 |  |  |  |  |  |
| *Excluding outlier, N = 59* | |  |  |  |  |  |  |  |
| Intercept | -0.314 | -1.223 | 0.594 |  |  |  |  |  |
| Faeces | 0.386 | -1.044 | 1.816 |  |  |  |  |  |
| Free Blood | 0.821 | -0.248 | 1.891 |  |  |  |  |  |
| Total Blood | 0.432 | -0.563 | 1.426 |  |  |  |  |  |
| *Moderator: sex* |  |  |  |  |  |  |  |  |
| *All data, N = 48* |  |  |  |  |  |  |  |  |
| Intercept | -0.188 | -0.861 | 0.486 |  |  |  |  |  |
| Male | 0.347 | -0.354 | 1.047 |  |  |  |  |  |
| *Excluding outlier, N = 47* | |  |  |  |  |  |  |  |
| Intercept | -0.068 | -0.692 | 0.557 |  |  |  |  |  |
| Male | 0.123 | -0.543 | 0.788 |  |  |  |  |  |
| *Moderator: Season* | | | |  |  |  |  |  |
| *All data, N = 52* |  |  |  |  |  |  |  |  |
| Intercept | 0.162 | -0.224 | 0.547 |  |  |  |  |  |
| Non-breeding | -0.033 | -0.588 | 0.521 |  |  |  |  |  |
| *Excluding outlier, N = 51* | |  |  |  |  |  |  |  |
| Intercept | 0.135 | -0.243 | 0.512 |  |  |  |  |  |
| Non-breeding | -0.015 | -0.561 | 0.532 |  |  |  |  |  |
| *Moderator: Population size* | | | |  |  |  |  |  |
| *All data, N = 68* |  |  |  |  |  |  |  |  |
| Intercept | -0.181 | -2.143 | 1.779 |  |  |  |  |  |
| Population size | 0.019 | -0.123 | 0.162 |  |  |  |  |  |
| *Excluding outlier, N = 67* | |  |  |  |  |  |  |  |
| Intercept | -0.536 | -2.476 | 1.404 |  |  |  |  |  |
| Non-breeding | 0.045 | -0.096 | 0.185 |  |  |  |  |  |

**Table S3**. Model coefficients and lower (L.) and upper (U.) 95% credible intervals (CI) for the effect (g) of the habitat, and for each of the moderators on baseline glucocorticoid levels of all taxa. The table on the right shows the heterogeneity explained by each of the random effects for the full intercept-only model.

| **Baseline glucocorticoid levels** | | |  |  |  |  |  |  |
| --- | --- | --- | --- | --- | --- | --- | --- | --- |
| **All taxa** |  |  |  |  |  |  |  |  |
|  | **Est** | **L. CI** | **U. CI** |  |  | ***I^2^* Est.** | **2.5 % CI** | **97.5 % CI** |
| *Intercept-only model* |  |  |  |  | Species ID | 0.152 | 0.089 | 0.233 |
| *All data, N = 108* |  |  |  |  | Study ID | 0 | 0 | 0 |
| Intercept | 0.068 | -0.135 | 0.272 |  | Study/Group | 0 | 0 | 0 |
| *Excluding outlier, N = 107* |  |  |  |  | Total | 0.765 | 0.721 | 0.806 |
| Intercept | 0.055 | -0.142 | 0.252 |  |  |  |  |  |
| *One effect size per group, N = 78* | |  |  |  |  |  |  |  |
| Intercept | 0.023 | -0.215 | 0.260 |  |  |  |  |  |
| *Moderator: lifestage (all taxa)* | | |  |  |  |  |  |  |
| *All data, N =108* |  |  |  |  |  |  |  |  |
| Intercept | 0.066 | -0.146 | 0.278 |  |  |  |  |  |
| Juvenile | 0.053 | -0.556 | 0.661 |  |  |  |  |  |
| *Excluding outlier, N = 107* |  |  |  |  |  |  |  |  |
| Intercept | 0.054 | -0.152 | 0.259 |  |  |  |  |  |
| Juvenile | 0.044 | -0.558 | 0.647 |  |  |  |  |  |
| *Moderator: hormone source (all taxa)* | | |  |  |  |  |  |  |
| *All data, N =105* |  |  |  |  |  |  |  |  |
| Intercept | 0.032 | -0.255 | 0.319 |  |  |  |  |  |
| Feather | -0.160 | -0.779 | 0.460 |  |  |  |  |  |
| Faeces | -0.095 | -0.544 | 0.354 |  |  |  |  |  |
| Free Blood | 0.374 | -0.154 | 0.903 |  |  |  |  |  |
| Hair | -0.004 | -0.686 | 0.679 |  |  |  |  |  |
| *Excluding outlier, N = 104* |  |  |  |  |  |  |  |  |
| Intercept | 0.006 | -0.271 | 0.283 |  |  |  |  |  |
| Feather | -0.156 | -0.767 | 0.455 |  |  |  |  |  |
| Faeces | -0.068 | -0.500 | 0.365 |  |  |  |  |  |
| Free Blood | 0.387 | -0.139 | 0.912 |  |  |  |  |  |
| Hair | 0.012 | -0.651 | 0.765 |  |  |  |  |  |
| *Moderator: taxa (only birds and mammals)* | | |  |  |  |  |  |  |
| *All data, N = 103* |  |  |  |  |  |  |  |  |
| Intercept | 0.035 | -0.233 | 0.303 |  |  |  |  |  |
| Mammals | -0.005 | -0.466 | 0.455 |  |  |  |  |  |
| *Excluding outlier, N = 102* |  |  |  |  |  |  |  |  |
| Intercept | 0.017 | -0.244 | 0.278 |  |  |  |  |  |
| Mammals | 0.011 | -0.437 | 0.459 |  |  |  |  |  |
| *Moderator: sex (+taxa)* |  |  |  |  |  |  |  |  |
| *All data, N = 83* |  |  |  |  |  |  |  |  |
| Intercept | -0.040 | -0.439 | 0.359 |  |  |  |  |  |
| Male | 0.069 | -0.333 | 0.470 |  |  |  |  |  |
| Mammals | 0.054 | -0.295 | 0.404 |  |  |  |  |  |
| *Excluding outlier, N = 82* |  |  |  |  |  |  |  |  |
| Intercept | -0.048 | -0.444 | 0.347 |  |  |  |  |  |
| Male | 0.057 | -0.339 | 0.454 |  |  |  |  |  |
| Mammals | 0.066 | -0.279 | 0.413 |  |  |  |  |  |
| *Moderator: season (+ taxa)(only adults)* | |  |  |  |  |  |  |  |
| *All data, N = 73* |  |  |  |  |  |  |  |  |
| Intercept | 0.096 | -0.206 | 0.398 |  |  |  |  |  |
| Non-breeding | -0.059 | -0.533 | 0.415 |  |  |  |  |  |
| Mammals | -0.259 | -0.904 | 0.385 |  |  |  |  |  |
| *Excluding outlier, N = 72* |  |  |  |  |  |  |  |  |
| Intercept | 0.069 | -0.222 | 0.362 |  |  |  |  |  |
| Non-breeding | -0.038 | -0.504 | 0.428 |  |  |  |  |  |
| Mammals | -0.249 | -0.859 | 0.362 |  |  |  |  |  |
| *Moderator: population size* | | |  |  |  |  |  |  |
| *All data, N =108* |  |  |  |  |  |  |  |  |
| Intercept | -0.519 | -1.941 | 0.903 |  |  |  |  |  |
| Population size | 0.045 | -0.062 | 0.151 |  |  |  |  |  |
| *Excluding outlier, N = 107* |  |  |  |  |  |  |  |  |
| Intercept | -0.711 | -2.106 | 0.684 |  |  |  |  |  |
| Population size | 0.058 | -0.047 | 0.163 |  |  |  |  |  |

**Table S4**. Model coefficients and lower (L.) and upper (U.) 95% credible intervals (CI) for the effect (g) of the habitat, and for each of the moderators used, on stress-induced glucocorticoid levels of all taxa and birds. The table on the right shows the heterogeneity explained by each of the random effects for the full intercept-only model.

| **Stress-induced glucocorticoid levels** | | |  |  |  |  |  |  |  |
| --- | --- | --- | --- | --- | --- | --- | --- | --- | --- |
| **All taxa** |  |  |  |  |  |  |  |  |  |
|  | **Est** | **L. CI** | **U. CI** |  |  | ***I^2^* Est.** | **2.5 % CI** | **97.5 % CI** | |
| *Intercept-only model* |  |  |  |  | Study ID | 0.042 | 0.019 | 0.073 |  |
| *All data, N = 54* |  |  |  |  | Study/Group | 0.045 | 0.027 | 0.068 |  |
| Intercept | -0.079 | -0.359 | 0.202 |  | Species ID | 0.073 | 0.029 | 0.130 |  |
| *Excluding outlier, N = 53* | |  |  |  | Total | 0.694 | 0.623 | 0.757 |  |
| Intercept | -0.112 | -0.365 | 0.141 |  |  |  |  |  |  |
| *One effect size per group, N = 39* | |  |  |  |  |  |  |  |  |
| Intercept | -0.089 | -0.381 | 0.201 |  |  |  |  |  |  |
| *Moderator: Population size*  *All data, N = 54* | |  |  |  |  |  |  |  |  |
| Intercept | -0.743 | -2.937 | 1.451 |  |  |  |  |  |  |
| Population size | 0.049 | -0.115 | 0.215 |  |  |  |  |  |  |
| *Excluding outlier, N =53* | |  |  |  |  |  |  |  |  |
| Intercept | -0.874 | -2.827 | 1.079 |  |  |  |  |  |  |
| Population size | 0.059 | -0.089 | 0.206 |  |  |  |  |  |  |
| **Birds only** |  |  |  |  |  |  |  |  |  |
| *Intercept-only model, N = 48* | |  |  |  |  |  |  |  |  |
| *All data* |  |  |  |  |  |  |  |  |  |
| Intercept | -0.209 | -0.530 | 0.113 |  |  |  |  |  |  |
| *Excluding outlier, N =47* | |  |  |  |  |  |  |  |  |
| Intercept | -0.231 | -0.524 | 0.063 |  |  |  |  |  |  |
| *One effect size per group, N =33* | |  |  |  |  |  |  |  |  |
| Intercept | -0.168 | -0.516 | 0.179 |  |  |  |  |  |  |
| *Moderator: Population size*  *All data, N = 48* | |  |  |  |  |  |  |  |  |
| Intercept | -1.483 | -3.810 | 0.844 |  |  |  |  |  |  |
| Population size | 0.096 | -0.079 | 0.271 |  |  |  |  |  |  |
| *Excluding outlier, N =47* | |  |  |  |  |  |  |  |  |
| Intercept | -1.485 | -3.642 | 0.673 |  |  |  |  |  |  |
| Population size | 0.095 | -0.068 | 0.257 |  |  |  |  |  |  |
| **Male birds only** | |  |  |  |  |  |  |  |  |
| *Moderator: Season, N = 29* | |  |  |  |  |  |  |  |  |
| *All data* |  |  |  |  |  |  |  |  |  |
| Intercept | -0.124 | -0.532 | 0.285 |  |  |  |  |  |  |
| Non-breeding | 0.305 | -0.523 | 1.133 |  |  |  |  |  |  |
| *Moderator: hormone source, N = 29* | |  |  |  |  |  |  |  |  |
| *All data* |  |  |  |  |  |  |  |  |  |
| Intercept | -0.079 | -0.521 | 0.363 |  |  |  |  |  |  |
| Free blood | -0.228 | -0.899 | 0.444 |  |  |  |  |  |  |

**Table S5**. *Ad hoc* analysis to explore for baseline glucocorticoid levels using only current measures obtained from blood samples from all taxa and only from adult birds (after removing other taxa and juveniles). Model coefficients and lower (L.) and upper (U.) 95% credible intervals (CI) for the effect (g) of the habitat and each of the moderators used.

| **Baseline glucocorticoid levels from blood** | | |  |
| --- | --- | --- | --- |
| **All taxa** |  |  |  |
|  | **Est** | **L. CI** | **U. CI** |
| *Intercept-only model, N = 60* |  |  |  |
| Intercept | -0.002 | -0.322 | 0.318 |
| *Moderator: hormone source, N = 60* | |  |  |
| Intercept | 0.041 | -0.349 | 0.431 |
| Free blood |  |  |  |
| **Adult birds only** | |  |  |
| *Intercept-only model, N = 52* |  |  |  |
| Intercept | 0.242 | -0.207 | 0.691 |
| *Moderator: hormone source, N = 52* | |  |  |
| Intercept | 0.161 | -0.297 | 0.618 |
| Free blood | 0.365 | -0.129 | 0.859 |
